# Expression and biological-clinical significance of *hTR*, *hTERT *and *CKS2 *in washing fluids of patients with bladder cancer

**DOI:** 10.1186/1471-2490-10-17

**Published:** 2010-10-04

**Authors:** Letizia Mezzasoma, Cinzia Antognelli, Chiara Del Buono, Fabrizio Stracci, Emanuele Cottini, Giovanni Cochetti, Vincenzo N Talesa, Ettore Mearini

**Affiliations:** 1 Department of Experimental Medicine, Division of Cell and Molecular Biology, University of Perugia, Via Del Giochetto 06122 Perugia, Italy; 2 Department of Medical-Surgical Specialties and Public Health, Division of Public Health University of Perugia, Via Del Giochetto 06122 Perugia, Italy; 3 Department of Medical-Surgical Specialties and Public Health, Division of Urology and Andrology, University of Perugia, Didactic and Scientific District of Terni, Santa Maria General Hospital, Italy

## Abstract

**Background:**

at present, pathogenesis of bladder cancer (BC) has not been fully elucidated. Aim of this study is to investigate the role of human telomerase RNA (*hTR*), human telomerase reverse transcriptase (*hTERT*) and CDC28 protein kinase regulatory subunit 2 (*CKS2*) in bladder carcinogenesis and their possible clinical significance;

**Methods:**

the transcript levels of *hTR*, *hTERT *and *CKS2 *were quantified by Real time reverse transcriptase chain reaction in exfoliated cells from bladder washings of 36 patients with BC and 58 controls. The statistical significance of differences between BC bearing patients and control groups, in the general as well as in the stratified analysis (superficial or invasive BC), was assessed by Student's t test. Non parametric Receiver Operating Characteristics analysis (ROC) was performed to ascertain the accuracy of study variables to discriminate between BC and controls. The clinical value of concomitant examination of *hTR*, *hTERT *and *CKS2 *was evaluated by logistic regression analysis;

**Results:**

a significant decrease in *hTR *and a significant increase in *hTERT *or *CKS2 *gene expression were found between BC bearing patients and controls, as well as in the subgroups analysis. The area under the curve (AUC) indicated an average discrimination power for the three genes, both in the general and subgroups analysis, when singularly considered. The ability to significantly discriminate between superficial and invasive BC was observed only for *hTR *transcript levels. A combined model including *hTR *and *CKS2 *was the best one in BC diagnosis;

**Conclusions:**

our results, obtained from a sample set particularly rich of exfoliated cells, provide further molecular evidence on the involvement of *hTR, hTERT *and *CKS2 *gene expression in BC carcinogenesis. In particular, while *hTERT *and *CKS2 *gene expression seems to have a major involvement in the early stages of the disease, *hTR *gene expression, seems to be more involved in progression. In addition, our findings suggest that the studied genes have a clinical role in discriminating between BC and controls in the general as well as in the stratified analysis, when singularly considered. A combined model improved over the single marker BC diagnosis.

## Background

Bladder cancer (BC) is one of the most common worldwide malignancies. In the Western world it is the fourth most common malignancy among men, following prostate, lung and colon cancers and represents the second most common cause of death among genitourinary tumors [1]. BC consists of a heterogeneous group of tumors that display a broad clinical spectrum ranging from superficial and well differentiated lesions to invasive and poorly differentiated cancers, which represents a key problem in its management. While there is a wealth of molecular information on BC, it is still not possible to derive a clear model for the molecular pathogenesis of all these tumors [2]. An enhanced understanding of the molecular biology of BC, could provide new insight into BC pathogenesis.

Telomerase is a specialized ribonucleoprotein complex including an RNA component, human telomerase RNA (*hTR*), and a catalytic protein, telomerase reverse transcriptase (*hTERT)*, which stabilizes the telomeres of linear chromosomes [3,4]. Although telomerase activity is present during human embryonic development, its expression and activity are repressed in most normal adult tissues. In contrast, most human tumours display high levels of telomerase [4-6]. Such an expression in cancer cells might be a necessary and essential step for tumor development and progression [6]. On the other hand, other findings indicate that telomerase expression might not be an obligate requirement in some settings for initial tumor growth, but play an important role for long-term maintenance [7,8]. Moreover, other observations suggest an additional role for telomerase during multistep oncogenesis [9]. In particular, further developments indicate that telomere biology knowledge still remains incomplete, and implicate additional complexity in the relationship among telomeres, telomerase and cancer [9].

The subunit 2 of the cyclin kinase Cdc28/CDC2 (*CKS2*) is an essential component for cell cycle control, involved in cell cycle progression from G1 to S and from G2 to M [10]. It has also been shown that *CKS2 *is essential for the first metaphase/anaphase transition of mammalian meiosis [11]. Accumulating evidence shows an extensive expression of *CKS2 *in malignant tumors of different tissues, including meningioma [12] as well as prostate [13], cervical [14], gastric [15], colon and liver [16] carcinomas.

The role of telomerase or *CKS2 *in carcinogenesis, has made these molecules of growing interest in BC research. Regarding the former, studies have pointed out that telomerase activity as well as the mRNA expression levels of its subunits are associated with malignancy in many BC tumor histotypes [17-22]. In particular, the expression of *hTERT *and *hTR *mRNA, both in tissues [22] and in voided urine samples [23], seems to correlate positively with tumor stage and grade, even if these data have not, as yet, been confirmed [24]. Hence, the biological relevance of telomerase remains to be fully elucidated and needs further investigation. Recently, *CKS2 *has been also studied in BC where it was significantly up-regulated, not only when BC was compared to normal bladder tissue, but also when invasive was compared to superficial BC [25]. The difference in the *CKS2 *expression level between invasive BC and the normal bladder tissue was greater than between superficial BC and the normal bladder tissue, thus suggesting that *CKS2 *expression may influence BC progression via cell cycle advancement [25]. At present, this is the only work to describe a possible role of *CKS2 *in this neoplasia.

The aim of this study was to investigate the biological role of *hTR*, *hTERT *and *CKS2 *in BC development and progression. Therefore, in the first part of the present study, we quantified the transcript levels of these molecules in samples particularly rich of exfoliated cells (bladder washings) from patients with or without BC.

Since we observed significant changes in the expression levels of the three considered genes between BC bearing patients and controls, we also decided to evaluate their possible role as molecular markers of BC diagnosis and progression.

## Methods

### Patients database

The present project was developed at the Cell and Molecular Biology Laboratory of the University of Perugia together with the Urology Service of the University Hospital. The study protocol followed the guidelines of our local ethics committee and the investigation was conducted with the ethical requirements defined in the Helsinki Declaration. All patients gave their informed consent to participate in the study. The study included 94 consecutive patients undergoing flexible cystoscopy either for bladder cancer (BC) diagnosis or for other clinical indications. The subjects were classified into two age and sex matched groups. The first one included 36 patients (32 male, 4 female) with a histopathological diagnosis of BC. All these patients were at the first diagnosis of BC. Mean age ± SD of BC group was 68.8 ± 10.8 years (range 48 to 87). The second group (controls) included 58 patients (49 male, 9 female) with a mean age ± SD of 69.9 ± 10.6 years (range 41 to 86). Tumor stage was determined using TNM (Tumor lymph Nodes and Metastasis) and grading according to the World Health Organization (WHO 2004) guide lines. All tumors were classified as: 72.2% (26/36) superficial low grade [pTa (n = 24), pT1 (n = 2)], 27.8% (10/36) muscle invasive high grade [pT2-4 (n = 10)]. Among controls 24.1% (14/58) were patients with no history of malignancy (with hematuria/irritative symptoms) and 75.9% (44/58) were patients enrolled in a 2 years follow up from the time of BC diagnosis. At the time of sampling, all controls were BC free.

### Collection of samples

60 ml of washing fluids were collected during flexible cystoscopy and immediately cooled on ice. Upon centrifugation at 4°C and 1200 rpm for 10 min, the sediments containing exfoliated cells were washed twice by suspension in ice-cold phosphate-buffered saline (PBS) and further centrifugation at 4°C and 1200 rpm for 10 min as well. Sediments thus obtained were snap frozen in TRIzol Reagent (Invitrogen, Milan, Italy), and stored at -70°C until subsequent use. We used bladder washes for detection of *hTERT*, *hTR *and *CKS2 *gene expression, because the number of exfoliated cells in these fluids has been shown to be higher than in voided urine [26]. In addition, the sensitivity of bladder washes in detection of urothelial malignancy has been shown to be better than voided urine [27]. Besides, cytology from bladder washing has been shown to be better than that from voided urine in the detection of bladder cancer [28,29].

### RNA isolation and cDNA synthesis

Total cellular RNA was isolated from the sediments using TRIzol Reagent (Invitrogen, Milan, Italy) according to the manufacturer's instructions. The quality and quantity of RNA was established spectrophotometrically by absorbance readings at 260 and 280 nm. Total RNA (1 μg) was reverse transcribed using the RevertAid™H Minus First Strand cDNA Synthesis Kit (Fermentas, Hanover, MD) and random primers System (Invitrogen, Milan, Italy). Following cDNA synthesis, the resulting mixture was heated at 95°C for 5 min before storage at -20°C.

### Quantitative Real Time PCR analysis

Beacon Designer 4 software (Stratagene, La Jolla, CA) was used for the design of suitable combinations, either of TaqMan primers and probes or SYBR Green primers. The sequences of oligonucleotide primers and probes used for real time PCR were as follows: human telomerase RNA (*hTR*): 5'-cgccttccaccgttcattc-3' (sense, 400 nM), 5'-gctgacagagcccaactc-3' (antisense, 400 nM), 5'-FAM-agctgctggcccgttcgccc-TAMRA-3' (TaqMan Probe, 200 nM); human telomerase reverse transcriptase (*hTERT*): 5'-cgagagcagacaccagcag-3'(sense, 400 nM), 5'-cggacactcagccttcagc-3'(antisense, 400 nM); CDC28 protein kinase regulatory subunit 2 (*CKS2*): 5'-catgagccagaaccacatattc-3'(sense, 400 nM), 5'-cagctcatgcacaggtatgg-3'(antisense, 400 nM); *β actin*: 5'-cactcttccagccttccttcc-3'(sense, 600 nM), 5'-acagcactgtgttggcgtac-3'(antisense, 600 nM), 5'-Cy5-tgcggatgtccacgtcacacttca-BHQ2-3'(TaqMan Probe, 200 nM). Standards were prepared by classical PCR from cDNA obtained from LNCaP cell line (ATCC # CRL-1740) for the concerned target mRNAs. PCR products were purified from agarose gel using the Qiaquick DNA Fragment Purification kit (Qiagen, Milan, Italy), according to the manufacturer's instructions. Serial dilutions of each standard were subsequently prepared to obtain, following real time PCR amplification, the reference standard curve to extrapolate quantitative information for cDNA targets of unknown concentrations. Detection of specific mRNAs expression was carried out by either quantitative Real Time TaqMan or SYBR Green PCR analysis on a MX3005P Real-Time PCR System (Stratagene, La Jolla, CA). The amplification reactions were performed in quadruplicate for each sample. In our experiments, the calibration curves consisted of at least 6 points, and each concentration was run in triplicate. Only calibration curves with an R square (R^2^) value of 0.985-0.995 and efficiency between 90% and 100% were considered. Each PCR run consisted of the specific 6 point calibration curve, a no template control, and the specimen cDNAs.

As to *CKS2*, *hTR*, *hTERT*, and *β actin *(the housekeeping gene used for normalization), PCR reactions were performed in a total volume of 25 μl, containing 250 ng of cDNA for *CKS2 *and 500 ng for *hTR *and *hTERT*, 1× Brilliant QPCR master mix or Brilliant SYBR Green QPCR Master mix (Stratagene, La Jolla, CA), plus a concentration of specific primers and probes, as above described. The PCR conditions were: *CKS2*: 1 cycle at 95°C for 10 min, 45 cycles at 95°C for 15 s, 63°C for 1 min; *hTR*: 1 cycle at 95°C for 10 min, 45 cycles at 95°C for 30 s, 61°C for 1 min; *hTERT*: 1 cycle at 95°C for 10 min, 45 cycles at 95°C for 30 s, 64°C for 1 min, 72°C for 30 s; *β actin*: 1 cycle at 95°C for 10 min, 45 cycles at 95°C for 30 s, 64°C for 1 min, 72°C for 30 s.

The level of *β actin *expression was measured in all samples to normalize *hTR*, *hTERT *and *CKS2 *expression for sample-to-sample differences in total volume of bladder washings, numbers of exfoliated cells, RNA input, RNA quality, and reverse transcription efficiency.

### Statistical Analysis

The results concerning the groups of patients with or without BC were compared by χ^2 ^test for categorical variables. The statistical significance of differences between BC patients and control groups was assessed by Student's t test. Differences were considered significant when P < 0.05. Most analyses were carried out using ln transformed variables to improve normality of distribution and data interpretability.

Nonparametric receiver operating characteristic analysis (ROC) was performed to assess the accuracy of study variables to discriminate between BC patients and controls [30,31]. Logistic regression was used to assess the independent predictive ability of study variables. The individual probabilities of a positive outcome, based on the model coefficients, were used to calculate the AUCs after logistic regression.

Due to the asymmetry and large variability of the observed urinary concentrations, logistic regression was performed on ln transformed data. The logistic model was calculated in the presence of only 5% of missing data, thus not leading to biased results. A multinomial logistic model was fitted to study data for the following three categories: controls (reference), superficial bladder cancer (SBC) and invasive bladder cancer (IBC). Stata 10 SE was used to perform statistical analyses (Stata Corp. 2007. Stata Statistical Software: Release 10. College Station, TX: StataCorp LP).

## Results

### Quantification of markers transcripts

**Figure 1:**
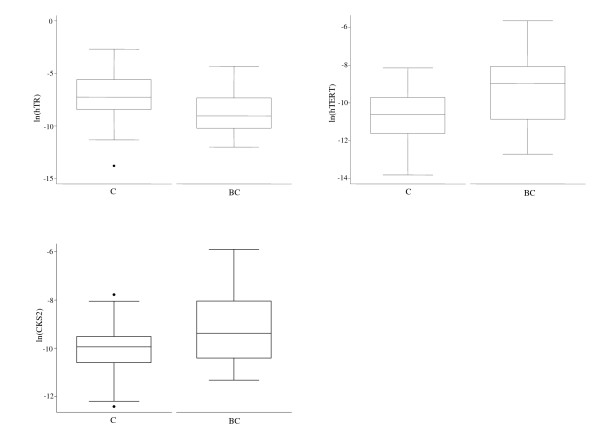
**Boxplots of mRNA expression levels of human Telomerase RNA (*hTR*), human Telomerase Reverse Transcriptase (*hTERT*) and CDC28 protein kinase regulatory subunit 2 (*CKS2*), measured by real time PCR in exfoliated cells from bladder washings of controls (C) and bladder cancer (BC) patients**. The median values are depicted as solid lines.

Table 1 shows the descriptive statistics of transcript levels concerning human telomerase RNA (*hTR*), human telomerase reverse transcriptase (*hTERT*) and CDC28 protein kinase regulatory subunit 2 (*CKS2*) in exfoliated cells from bladder washings carried out in bladder cancer (BC) patients and controls. Real time PCR analysis of the three studied genes revealed highly significant differences between BC and controls. In particular, *hTR *showed a significant 4.4 fold decrease compared to controls (Figure 1). Conversely, *hTERT *and *CKS2 *showed a significant 11.4 fold and 5.6 fold increase compared to controls, respectively (Figure 1).

**Table 1:** Mean (± SE), 95% Confidence Interval (CI), median values for main study variables.

	C			BC		
**Variable**	**Mean (± SE)**	**95% CI**	**Median**	**Mean (± SE)**	**95% CI**	**Median**

*hTR*	3.9 × 10^-3 ^(±1.3 × 10^-3^)	1.3 × 10^-3 ^-6.5 × 10^-3^	7.0 × 10^-4^	8.9 × 10^-4 ^(±4.2 × 10^-4^)	3.5 × 10^-5 ^-1.7 × 10^-3^	1.2 × 10^-4^
*hTERT*	4.2 × 10^-5 ^(±8.5 × 10^-6^)	2.4 × 10^-5 ^-5.9 × 10^-5^	2.0 × 10^-5^	4.8 × 10^-4 ^(±1.5 × 10^-4^)	1.7 × 10^-4 ^-7.8 × 10^-4^	1.2 × 10^-4^
*CKS2*	6.1 × 10^-5 ^(±9.2 × 10^-6^)	4.2 × 10^-5 ^-7.9 × 10^-5^	4.8 × 10^-5^	3.4 × 10^-4 ^(±1.0 × 10^-4^)	1.3 × 10^-4 ^-5.5 × 10^-4^	8.4 × 10^-5^

C: Controls; BC: Bladder Cancer; *hTR*: human Telomerase RNA; *hTERT*: human Telomerase Reverse Transcriptase; *CKS2*: CDC28 protein kinase regulatory subunit 2; CI: Confidence Interval; SE: Standard Error.

**Figure 2:**
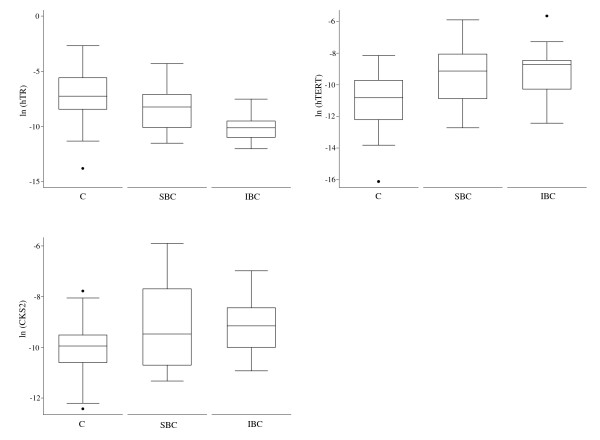
**Boxplots of mRNA expression levels of human Telomerase RNA (*hTR*), human Telomerase Reverse Transcriptase (*hTERT*) and CDC28 protein kinase regulatory subunit 2 (*CKS2*), measured by real time PCR in exfoliated cells from bladder washings of controls (C) and superficial bladder cancer (SBC) and invasive bladder cancer (IBC) patients**. The median values are depicted as solid lines.

In the attempt to evaluate a possible role of the studied genes in the development and progression of bladder tumors, we compared the transcript levels of *hTR*, *hTERT *and *CKS2 *in exfoliated cells from superficial low grade (pTa, pT1) or muscle invasive high grade (pT2-4) BC and controls (Table 2). All analyses comparing sub-groups by stage are exploratory, because of the small number of study cases. We found significant differences in the expression levels of the three considered genes among the control, superficial and invasive groups. In particular, regarding *hTR*, a significant decrement was observed in superficial BC (SBC), becoming more evident in invasive BC (IBC) (Figure 2). Conversely, *hTERT *and *CKS2 *showed almost the same up regulation both in SBC and IBC (Figure 2).

**Table 2:** Mean (± SE), 95% Confidence Interval (CI), median values for main study variables.

	C			SBC			IBC		
**Variable**	**Mean (± SE)**	**95% CI**	**Median**	**Mean (± SE)**	**95% CI**	**Median**	**Mean (± SE)**	**95% CI**	**Median**

*hTR*	3.9 × 10^-3 ^ (±1.3 × 10^-3^)	1.3 × 10^-3^-6.5 × 10^-3^	7.03 × 10^-4^	1.2 × 10^-3 ^ (±5.6 × 10^-4^)	1.5 × 10^-5^-2.3 × 10^-3^	2.6 × 10^-4^	9.3 × 10^-5 ^ (±5.6 × 10^-5^)	-3.7 × 10^-5^-2.2 × 10^-4^	4.0 × 10^-5^
*hTERT*	4.2 × 10^-5 ^ (±8.5 × 10^-6^)	2.4 × 10^-5^-5.9 × 10^-5^	2 × 10^-5^	4.7 × 10^-4 ^ (±1.7 × 10^-4^)	1.2 × 10^-4^-8.1 × 10^-4^	1.1 × 10^-4^	5.0 × 10^-4 ^ (±3.4 × 10^-4^)	-2.6 × 10^-4^-1.3 × 10^-3^	1.7 × 10^-4^
*CKS2*	6.1 × 10^-5 ^ (±9.2 × 10^-6^)	4.2 × 10^-5^-7.9 × 10^-5^	4.8 × 10^-5^	3.8 × 10^-4 ^ (±1.4 × 10^-4^)	1.0 × 10^-4^-6.7 × 10^-4^	7.7 × 10^-5^	2.1 × 10^-4 ^ (±9.6 × 10^-5^)	-1.1 × 10^-5^-4.3 × 10^-4^	1.1 × 10^-4^

C: Controls; SBC: Superficial Bladder Cancer; IBC: Invasive Bladder Cancer; *hTR*: human Telomerase RNA; *hTERT*: human Telomerase Reverse Transcriptase; *CKS2*: CDC28 protein kinase regulatory subunit 2; CI: Confidence Interval; SE: Standard Error.

### hTR, hTERT and CKS2 as molecular markers of bladder cancer

**Figure 3:**
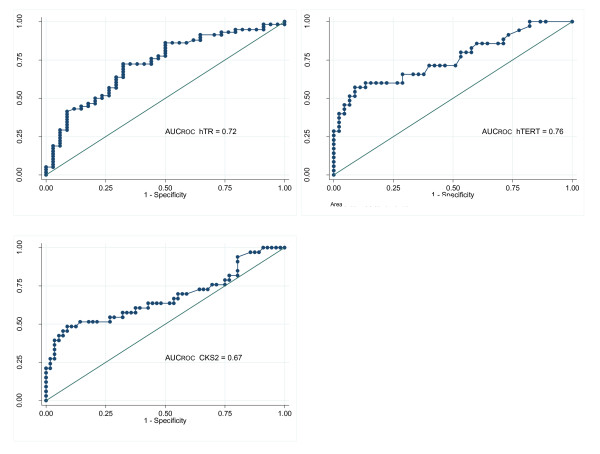
**ROC curves and AUCs for human Telomerase RNA (*hTR*), human Telomerase Reverse Transcriptase (*hTERT*) and CDC28 protein kinase regulatory subunit 2 (*CKS2*)**.

In the attempt to evaluate the ability of each considered molecule in discriminating between bladder cancer (BC) and controls, we performed Receiver Operating Characteristic (ROC) curves for each study variable. The area under the curve (AUC) was 0.72 (95% CI: 0.62-0.83) for *hTR*, 0.76 (95% CI: 0.65-0.87) for *hTERT *and 0.67 (95% CI: 0.55-0.80) for *CKS2*, thus indicating an average discrimination power between BC and controls for all these tests, when singularly considered (Figure 3).

**Figure 4:**
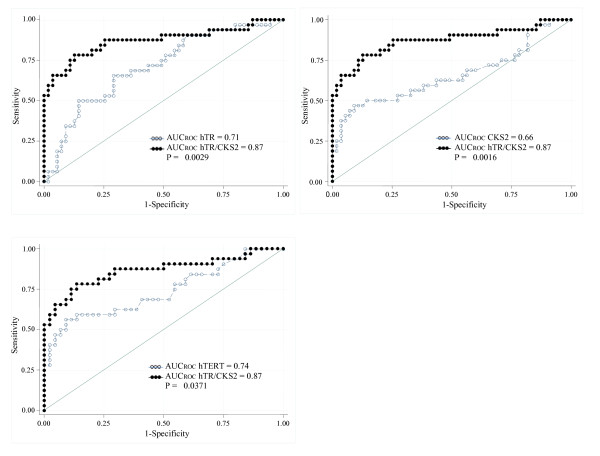
**ROC curves derived from the model including human Telomerase RNA (*hTR*) and CDC28 protein kinase regulatory subunit 2 (*CKS2*) compared with *hTR*, *CKS2 *and human Telomerase Reverse Transcriptase (*hTERT*) ROC curves**.

In order to establish if a combination of markers could enhance the diagnostic relevance of the assay, we evaluated, by using a logistic regression model, whether the whole of all 3 markers, combinations of 2 of them, or the use of single ones were most useful. A model including *hTR *and *CKS2 *was the best one in BC diagnosis (Table 3) and showed a higher clinical performance in comparison to each single tested marker (AUC*_hTR _*= 0.71, 95% CI: 0.60-0.82; AUC*_hTERT _*= 0.74, 95% CI: 0.625-0.86; AUC*_CKS2 _*= 0.66, 95% CI: 0.53-0.79; AUC*_hTR/CKS2 _*= 0.87, 95% CI: 0.78-0.96) (Figure 4).

**Table 3:** Logistic regression model for BC diagnosis.

**Predictors**	**OR**	**P**	**95% CI**
	
ln(*hTR*)	0.43	0.0001	0.30-0.63
ln(*CKS2*)	4.2	0.0001	2.13-8.30
AUC_ROC_	0.87		0.78-0.96

BC: Bladder Cancer; *hTR*: human Telomerase RNA; *CKS2*: CDC28 protein kinase regulatory subunit 2; OR: Odds Ratio; 95% Confidence Interval; AUC: Area Under Curve; ROC: Receiver Operating Characteristic curve.

In the attempt to evaluate the ability of each considered molecule in discriminating between SBC or IBC and controls, we performed ROC curves for each study variable. The AUCs values were 0.67 (95% CI: 0.56-0.77) for *hTR*, 0.75 (95% CI: 0.63-0.84) for *hTERT *and 0.65 (95% CI: 0.54-0.76) for *CKS2*. Such results pointed out an average discrimination power between controls and superficial forms for these tests, when singularly considered. The same analysis was performed for invasive forms. The AUCs values were 0.88 (95% CI: 0.78-0.95) for *hTR*, 0.78 (95% CI: 0.65-0.88) for *hTERT *and 0.72 (95% CI: 0.60-0.82) for *CKS2*. Finally, the ability to significantly discriminate between superficial and invasive BC was evaluated only for *hTR *transcript level (AUC: 0.78, 95% CI: 0.60-0.90, P = 0.0005). Therefore, all the three studied molecules appear to be suitable in discriminating superficial or invasive BC forms from controls. In addition, *hTERT *was the best one in discriminating BC superficial forms, while *hTR *was the best one in discriminating BC invasive forms.

To explore if the use of more predictors would be able to discriminate also between controls and SBC or IBC, a multinomial logistic model was employed. The *hTR*/*CKS2 *combined model showed an improved discrimination between controls and SBC or IBC with respect to each marker alone (Table 4).

**Table 4:** Multinomial logistic model comparing controls (C, reference) with superficial (SBC) and invasive bladder cancer (IBC).

**Predictors**	**OR**	**P**	**95% CI**
	
**C**	**Ref**.		
	
**SBC**			
ln(*hTR*)	0.50	0.0001	0.34-0.74
ln(*CKS2*)	3.9	0.0001	1.95-7.76
	
**IBC**			
ln(*hTR*)	0.10	0.001	0.03-0.41
ln(*CKS2*)	16.7	0.001	3.19-87.2
	
AUC_ROC[C-SBC]_	0.82		0.71-0.94
AUC_ROC[C-IBC]_	0.98		0.95-1.0

*hTR*: human Telomerase RNA; *CKS2*: CDC28 protein kinase regulatory subunit 2; OR: Odds Ratio; 95% Confidence Interval; AUC: Area Under Curve; ROC: Receiver Operating Characteristic curve.

## Discussion

Bladder tumors show widely differing histopathology and clinical behavior. During the past 10 years, evidence has accrued on molecular pathways of bladder cancer (BC). However, molecular mechanisms of BC development and progression are not fully understood.

Our study characterizes the expression levels of three different genes associated with carcinogenesis: human telomerase RNA (*hTR*), human telomerase reverse transcriptase (*hTERT*) and CDC28 protein kinase regulatory subunit 2 (*CKS2*) in BC patients and controls. The evaluation was made in sediments from bladder washings, samples particularly rich of exfoliated tumor cells [26]. The choice of using bladder washings is related to their usefulness and sensitivity in detecting urothelial malignancy, as it was previously shown [27,28].

Our results point out a significant difference in the transcript levels of *hTR*, *hTERT *and *CKS2 *between BC and controls. In particular, when BC group was compared to controls, the former clearly showed a significant 4.4 fold decrease in *hTR *expression level. Such a result could be ascribed to specific regulation mechanisms at transcriptional level. In fact, transcriptional regulation is emerging as the main action controlling *hTR *gene expression [4]. Multiple mechanisms regulate the *hTR *promoter in vivo [32] and a number of transcription factors have been implicated. In particular, in bladder cancer cells, a role for MDM2 in *hTR *promoter regulation, has been recently demonstrated [33]. MDM2 associates with the *hTR *promoter and negatively regulates its activity [33], likely interfering with more than one transcriptional regulator in a dominant fashion [4].

The decrement in *hTR *expression, observed in our study, suggest its involvement in BC carcinogenesis. Until the present, studies in the literature described an increased expression of *hTR *in cancer patients with respect to healthy individuals [22,23]. However, a peer comparison with such studies is quite difficult to interpret. In fact, to our knowledge, they refer to the evaluation of *hTR *mRNA levels in urine samples and the only study evaluating the expression of this molecule in washing fluids is not methodologically comparable [27].

Conversely, *hTERT *expression levels showed an 11.4 fold increment in BC group compared to controls, suggesting that its up regulation may have an important role in BC carcinogenesis. The observed *hTERT *over expression, could reflect the necessity in producing high levels of proteins required for its biological function. In fact, a significant association of telomerase activity with *hTERT *expression has been already previously shown [26,34]. Such a higher expression in bladder washing fluids is in agreement with other previous findings [22-24,26,34-38].

With respect to *CKS2*, we observed a significant 5.6 fold up regulation in BC patients compared to controls, suggesting that aberrantly expressed *CKS2 *may contribute to BC initiation. Such a higher expression in bladder washing fluids is in agreement with the only report describing that *CKS2 *expression is strongly correlated with BC tumorigenesis [25].

Previous studies have shown that overexpression of cyclins D1 and E is associated with high levels of telomerase activity [39] and that *CDK *overexpression is required for telomerase activity in human and mouse cancer cells [40]. Therefore, the parallel overexpression of *hTERT *and *CKS2*, observed in our study, could suggest a similar correlation also in BC and their involvement in a common regulatory pathway.

We then evaluated the mRNA expression level of *hTR*, *hTERT *and *CKS2 *in superficial and invasive BC compared to controls. With regard to *hTR*, a strong down regulation correlating with BC progression was observed, suggesting a possible role in the evolution of the disease.

Conversely, *hTERT *and *CKS2 *transcript levels were significantly up regulated in superficial forms, remaining almost unchanged in the invasive ones, thus suggesting a possible involvement of both of them in the early events during tumor development. There have been several attempts to correlate *hTERT *expression with BC staging and histological grading often with rather conflicting results [22] or with results that have not, as yet, been confirmed [21]. The present study, provides further evidence supporting that *hTERT *mRNA expression is not related to tumor stage.

Regarding *CKS2*, the only report on this subject correlated *CKS2 *expression at tissue level, with tumor stage [25]. The discrepancy between this study and ours may be due to the different analyzed specimen. In fact, the results emerging from tissue analysis, are not always paralleled by the same significance in other samples.

Although the field of tumor markers in BC is rapidly evolving no ideal marker currently exists. Among the innovative methods of detection, the employ of molecular markers is promising. Molecular assays usually produce more qualitative (categorical) results with higher sensitivity and reproducibility than the continuous data typically produced by biochemical assays [41,42].

Since we observed significant changes in the expression levels of the three considered genes between BC and controls, we then evaluated their possible role as molecular markers of BC diagnosis and progression.

Calculating the Area Under the Receiver Operating Characteristic (ROC) curve (AUC), we assessed the discriminative ability of the transcript level concerning *hTR*, *hTERT *and *CKS2 *between BC and controls, when singularly considered (ROC areas ranging between 0.67-0.76). Subgroup analysis of the disease revealed that *hTR*, *hTERT *and *CKS2 *were able to discriminate between controls and superficial or invasive BC (AUCs ranging from 0.65 to 0.88).

Finally, our results suggest that a panel of markers, evaluated through the transcription of their genes, can be more useful than a single test for the diagnosis of BC. In particular, the combination of transcript levels of both *hTR *and *CKS2 *genes in the sediments of bladder washings, improves, over the single biomarker, BC diagnosis (AUC *_hTR/CKS2 _*0.87 vs AUCs ranging from 0.66 to 0.74). Hence, we agree with the general concept that for the diagnosis of heterogeneous diseases such as BC, the employment of a combined analysis of several markers seems to be the most promising approach [43].

With regard to subgroups analysis, the *hTR*/*CKS2 *combined model turned out to be useful in discriminating both superficial or invasive forms, compared to control. However, given the small number of invasive cancers, this may be regarded as an exploratory analysis.

## Conclusions

Our results, obtained from a sample set particularly rich of exfoliated cells, provide further molecular evidence on the involvement of *hTR, hTERT *and *CKS2 *gene expression in bladder cancer (BC) carcinogenesis. In particular, while *hTERT *and *CKS2 *gene expression seems to have a major involvement in the early pathogenesis of the disease, *hTR *gene expression seems to be more associated with BC progression. Furthermore, the investigation of a possible clinical role of the three considered genes points out the ability to generally discriminate between control and BC, or superficial or invasive BC, when singularly considered. Finally, our results suggest that a panel of markers, evaluated through the transcription of their genes, can be more useful than a single test for diagnosis of BC. In particular, the combination of bladder washings transcript levels of both *hTR *and *CKS2 *genes improves, over the single biomarker, BC diagnosis. Further investigation will be necessary to confirm the role of *hTR *in BC progression.

Therefore, it could be of particular interest to extend the study to a larger population and to confirm these results in urine, to provide a useful non-invasive tool in detection and clinical evaluation of BC.

## Competing interests

The authors declare that they have no competing interests.

## Authors' contributions

The work presented here was carried out in collaboration between all authors.

LM conceived of, designed and coordinated the study, analyzed the data and drafted the manuscript. CA participated in the design of the study, analyzed the data and drafted the manuscript. CDB carried out the laboratory experiments. FS performed the statistical analysis. GC and EC enrolled patients and provided clinical information. EM and VNT defined the research theme and critically revised the paper for important intellectual content.

All authors read and approved the final manuscript.

## Pre-publication history

The pre-publication history for this paper can be accessed here:

http://www.biomedcentral.com/1471-2490/10/17/prepub
